# Supplementary material for: Sex differences in alcohol use patterns and related harms: A mixed-methods, cross-sectional study of men and women in northern Tanzania
Source: PLOS Glob Public Health. 2024 Nov 21;4(11):e0003942. doi: 10.1371/journal.pgph.0003942 (PMC11581317; doi:10.1371/journal.pgph.0003942)
Supplement: S1 Table — (PDF) [file pgph.0003942.s001.pdf]

S1 Table

Itemized AUDIT scores by sample and sex

	Community (n = 500) (%)	Men (n = 223) (%)	Women (n = 277) (%)	Patient (n = 345) (%)	Men (n = 280) (%)	Women (n = 64) (%)	Men (n = 504) (%)	Women (n = 341) (%)	p-value[1]
**How often do you have a drink containing alcohol?**									
Never	1.1	0.7	1.6	2.01	1.875	2.56	42	52	<0.001
Monthly	31.7	19.3	44.9	12.06	10	20.51	8.3	19	<0.001
2–4 per month	21	24.4	17.3	25.13	23.75	30.76	14	10	<0.001
2–3 per week	24.8	29.6	19.7	37.69	38.13	35.89	20	11	<0.001
4+ per week	21.4	25.9	16.5	23.12	26.25	10.25	15	7.3	<0.001
**How many drinks containing alcohol do you have on a typical day when you are drinking?**									
1 or 2	52.3	38.5	66.9	41.5	35	66.67	63	84	<0.001
3 or 4	35.9	48.1	22.8	41.5	45	28.21	27	12	<0.001
5 or 6	6.1	8.1	3.9	10	11.25	5.13	5.8	2.1	<0.001
7, 8, or 9	3.1	3	3.1	4	5	0	2.4	1.2	<0.001
10+	2.7	2.2	3.1	3	3.75	0	1.8	1.2	<0.001
**How often do you have six or more drinks on one occasion?**									
Never	66.4	58.5	74.8	68.5	65	84.6	78	89	<0.001
Less than monthly	9.5	10.4	8.7	8	8.75	5.13	5.6	3.8	<0.001
Monthly	14.1	18.5	9.4	8.5	8.13	7.69	7.7	4.4	<0.001
Weekly	6.5	8.9	3.9	8	9.38	2.56	5.4	1.8	<0.001
Daily or almost daily	3.4	3.7	3.1	7	8.75	0	3.8	1.2	<0.001
**How often during the last year have you found that you were not able to stop drinking once you started?**									
Never	76.7	65.9	88.2	66	60	89.74	78	94	<0.001
Less than monthly	7.6	10.4	4.7	7.5	8.75	2.56	5.6	2.1	<0.001
Monthly	7.3	11.1	3.1	7.5	8.75	2.56	5.8	1.5	<0.001
Weekly	4.6	7.4	1.6	12	14.38	2.56	6.5	0.9	<0.001
Daily or almost daily	3.8	5.2	2.4	7	8.13	2.56	4	1.2	<0.001
**How often during the last year have you failed to do what was normally expected from you because of drinking?**									
Never	84.7	79.3	90.6	82	79.38	92.3	88	96	0.002
Less than monthly	4.2	5.9	2.4	2.5	3.13	0	2.6	0.9	0.002

Monthly	5.7	6.7	4.7	5.5	6.25	2.56	3.8	2.1	0.002
Weekly	2.7	5.2	0	5	5.63	2.56	3.2	0.3	0.002
Daily or almost daily	2.7	3	2.4	5	5.63	2.56	2.6	1.2	0.002
**How often during the last year have you needed a drink in the morning to get yourself going after a heavy drinking session?**									
Never	78	70.4	85.8	79.5	76.25	94.9	84	94	<0.001
Less than monthly	7.6	9.6	5.5	4	5	0	4.2	2.1	<0.001
Monthly	5.7	9.6	1.6	3.5	3.75	0	4	0.6	<0.001
Weekly	3.4	3.7	3.1	6.5	7.5	2.56	3.4	1.5	<0.001
Daily or almost daily	5.3	6.7	3.9	6.5	7.5	2.56	4.2	1.8	<0.001
**How often during the last year have you had a feeling of guilt or remorse after drinking?**									
Never	62.2	51.1	74	48	43.75	66.67	69	87	<0.001
Less than monthly	9.2	12.6	5.5	7	8.13	2.56	6	2.3	<0.001
Monthly	9.5	14.1	4.7	13	14.38	7.69	8.3	2.6	<0.001
Weekly	4.2	6.7	1.6	12.5	12.5	12.82	5.8	2.1	<0.001
Daily or almost daily	14.9	15.6	14.2	19.5	21.25	10.25	11	6.5	<0.001
**How often during the last year have you been unable to remember what happened the night before because you had been drinking?**									
Never	85.1	77.8	92.9	78.5	74.38	94.9	86	97	<0.001
Less than monthly	4.6	5.9	3.1	3	3.75	0	2.8	1.2	<0.001
Monthly	4.9	8.1	1.6	7	8.75	0	5	0.6	<0.001
Weekly	2.7	4.4	0.7	6.5	8.13	0	3.8	0.3	<0.001
Daily or almost daily	2.7	3.7	1.6	5	5	5.1	2.6	1.2	<0.001
**Have you or someone else been injured as a result of your drinking?**									
No	91.2	87.4	94.2	79.4	78.4	86.04	86	94	0.001
Yes, but no in the last year	5.6	8.1	3.6	12.1	12.8	6.97	8.9	3.8	0.001
Yes, during the last year	3.2	4.5	2.2	8.5	8.8	6.97	2.6	2.6	0.001
**Has a relative or friend or doctor or another health worker been concerned about your drinking or suggested you cut down?**									
No	70.8	57.8	81.2	63.3	60.8	76.7	65	82	<0.001
Yes, but no in the last year	17.6	25.1	11.6	23.4	25	13	21	11	<0.001
Yes, during the last year	11.6	17	7.2	13.3	14.2	9.3	13	7	<0.001

[1] unpaired t tests
