# Supplementary material for: Sex differences in alcohol use patterns and related harms: A mixed-methods, cross-sectional study of men and women in northern Tanzania
Source: PLOS Glob Public Health. 2024 Nov 21;4(11):e0003942. doi: 10.1371/journal.pgph.0003942 (PMC11581317; doi:10.1371/journal.pgph.0003942)
Supplement: S2 Table — (PDF) [file pgph.0003942.s002.pdf]

S2 Table

Itemized DrInC scores by sample and sex

	Community (n = 500) (%)	Men (n = 223) (%)	Women (n = 277) (%)	Patient (n = 345) (%)	Men (n = 280) (%)	Women (n = 64) (%)	Men (n = 504) (%)	Women (n = 341) (%)	p-value[1]
**Physical consequences**									
I have had a hangover or felt bad after drinking	42.1	59.4	27.5	37.8	42.1	16.3	38	19	<0.001
After drinking, I have had trouble with sleeping, staying asleep, or nightmares	13.9	19.4	9.2	14.2	15.8	6.9	13	6.5	0.003
I have been sick and vomited after drinking	17.8	21.2	15	20.8	22.9	9.3	17	10	0.012
Because of my drinking, I have not eaten properly	23	30.9	16.4	25.3	28.4	9.3	22	11	<0.001
My physical health has been harmed by my drinking	9.2	12.6	6.3	13.4	15.8	2.3	11	4.1	<0.001
My physical appearance has been harmed by my drinking	8	12.6	4.3	13.4	15.8	2.3	11	2.9	<0.001
My sex life suffered because of my drinking	13.6	24.6	4.3	15.4	17.8	4.6	16	3.2	<0.001
While drinking ot intoxicated, I have been physically hurt, injured, or burned	6.8	12	2.4	15.4	17.2	6.9	11	2.3	<0.001
**Intrapersonal consequences**									
I have felt bad about myself because of my drinking	34.9	54.6	18.4	38.2	42.6	16.3	36	13	<0.001
I have been unhappy because of my drinking	23.8	34.3	15	24.5	26.9	11.6	23	11	<0.001
I have felt guilty or ashamed because of my drinking	21.7	34.9	10.6	25.3	26.9	16.3	23	8.5	<0.001
When drinking, my personality has changed for the worse	14	22.9	6.3	19.2	21.3	9.5	16	5	<0.001
I have lost interest in activities and hobbies because of my drinking	12.4	21.7	6.3	17.1	19.3	6.9	15	4.7	<0.001
My spiritual or moral life has been harmed by my drinking	13.1	21.1	6.3	5.2	28	0	19	5	<0.001
Because of my drinking, I have not had the kind of life that I want	23	34.9	13	29.4	32.8	13.9	25	9.7	<0.001
My drinking has gotten in the way of my growth as a person	51.3	57.1	46.4	66.1	66.2	65.1	46	36	0.005
**Social responsibility consequences**									
I have missed days of work or school because of my drinking	8.4	16.7	1.4	18.7	21.8	4.6	14	1.5	<0.001
The quality of my work has suffered because of my drinking	10.2	18.3	3.4	17.1	19.8	2.3	14	2.3	<0.001
I have failed to do what is expected of me because of my drinking	21.2	33.1	11.1	22.4	25.2	6.9	22	7.6	<0.001
I have gotten into trouble because of drinking	19.4	28.6	11.6	24.1	26.9	11.6	21	8.5	<0.001

I have had money problems because of my drinking	24.3	37.7	13	30.7	35	11.6	27	9.4	<0.001
I have spent too much or lost a lot of money because of my drinking	28	42.3	15.9	33.7	38.6	11.6	30	11	<0.001
I have been suspended/fired from or left a job or school because of my drinking	3.2	5.7	0.98	6.5	7.4	2.3	5	0.9	<0.001
**Interpersonal consequences**									
My family or friends have worried or complained about my drinking	22	35.4	10.6	26.8	30.7	6.9	25	7.3	<0.001
My ability to be a good parent has been harmed by my drinking	12.6	18.9	7.3	16.3	18.3	4.6	14	5	<0.001
While drinking, I have said or done embarrassing things	12	21.1	4.3	15.9	17.3	9.3	14	3.8	<0.001
While drinking or using drugs, I have said harsh or cruel things to someone	1.3	2.9	0	8.9	10.4	2.3	5.2	0.3	<0.001
My marriage or love relationship has been harmed by my drinking	12.8	20.6	6.3	17.9	20.3	6.9	15	4.7	<0.001
My family has been hurt by my drinking	18.1	28	9.7	21.9	25.7	4.6	20	6.5	<0.001
A friendship or close relationship has been damaged by my drinking	7.3	12	3.4	14.5	16.7	4.6	11	2.6	<0.001
My drinking has damaged my social life, popularity, or reputation	12.6	20	6.3	22	24.8	9.3	17	5	<0.001
I have lost a marriage or a close love relationship because of my drinking	9.4	15.4	4.3	12.2	14.4	2.3	11	2.9	<0.001
I have lost a friend because of my drinking	5.2	9.1	1.9	11.3	13.3	2.3	8.5	1.5	<0.001
**Impulse control consequences**									
I have driven a motor vehicle after having three or more drinks	12.8	24	3.4	6.9	8.4	0	12	2.1	<0.001
My drinking has caused me to use other drugs more	2.6	5.1	0.4	5.3	5.94	0	4.4	0.3	<0.001
I have taken foolish risks when I have been drinking	25.4	36	16.4	26	28.2	16.3	24	12	<0.001
When drinking, I have done impulsive things that I regretted later	20.4	32.6	10.1	18.4	21.4	4.6	20	6.7	<0.001
I have gotten into a physical fight while drinking	7.3	13.7	1.9	19.1	22.3	4.6	14	1.8	<0.001
I have had an accident while drinking or intoxicated	6.3	12	1.4	15.4	18.2	2.3	12	1.2	<0.001
While drinking or intoxicated, I have injured someone else	5	9.7	0.97	9.3	10.4	4.6	7.5	1.2	<0.001
I have broken things while drinking or intoxicated	2.9	6.2	0	9.8	10.9	4.6	6.5	0.6	<0.001
I have smoked tobacco more when I am drinking	11	21.7	1.9	16.3	19.3	2.3	15	1.5	<0.001
I have been overweight because of my drinking	6.5	8	5.3	4.1	4.95	0	4.8	3.2	0.4

I have been arrested for driving under the influence of alcohol	4.5	8.6	0.96	4.1	5	0	5	0.6	<0.001
I have had trouble with the law (other than driving while intoxicated) because of my drinking	4.2	8	0.96	6.9	7.9	2.3	6	0.9	<0.001
**Control items**									
I have enjoyed the taste of beer, wine, or liquor	80.9	84	78.3	45.9	43.6	55.8	46.6	54.5	<0.001
Drinking has helped me to relax	29.3	37.7	22.2	22.1	23	16.3	22.2	15.5	<0.001
Drinking has helped me to have a more positive outlook on life	6	9.7	2.9	6.5	7.9	0	6.5	1.8	<0.001
When drinking, my social life has been more enjoyable	14.1	18.3	10.6	11	10.4	13.9	8.5	23.2	<0.001
I drank alcohol normally, without any problems	62.3	52.6	70.5	47	46.3	48.8	36.9	48.9	<0.001

[1]unpaired t tests
