# Supplementary material for: Sex differences in alcohol use patterns and related harms: A mixed-methods, cross-sectional study of men and women in northern Tanzania
Source: PLOS Glob Public Health. 2024 Nov 21;4(11):e0003942. doi: 10.1371/journal.pgph.0003942 (PMC11581317; doi:10.1371/journal.pgph.0003942)
Supplement: S1 Appendix — (PDF) [file pgph.0003942.s003.pdf]

S1 APPENDIX

Focus group semi-structured discussion guide

1. When are people in Moshi first offered alcohol?
2. Who are those who first offer alcohol to others? Friends, family?
3. How available is alcohol to children? To teenagers? To young adults? To the elderly?
4. How costly is alcohol, and does alcohol decrease the availability to potential alcohol users?
5. Is there a stigma associated with moderate alcohol use? With heavy alcohol use? With seeking care for alcohol? With being intoxicated? With being intoxicated in public? With alcohol use while operating a car or motorcycle?
6. Are there any friends or family you know that have had a problem with drinking? Have they ever sought any help, guidance or treatment? What kinds of treatments are available?
7. Are there differences in when and where women commonly drink compared to men?
    a. Are there differences in the amount of alcohol consumed between men and women?
8. Are women who drink alcohol viewed differently than men who drink alcohol?
    a. Is it more acceptable for men or women to drink?
9. What can someone who wants to stop drinking do to stop? Where can he/she get help?
    a. If anyone, who should he/she reach out to?
10. If you had a drinking problem would you report your accurate alcohol use to someone else if they had the ability to help you?
    a. Who would you feel most comfortable talking to about your alcohol use?
    b. Would you tell a nurse or a doctor?
    c. Do you think that someone’s family could give a more honest report of alcohol use than the patient? (only in patient relative FGD)
    d. Do you think a patient could give a more honest report of alcohol use than their family? (only in injury patient FGD)
11. What factors keep you from drinking and driving?
    a. Are you scared of the police, of getting injured, or is it something else?
    b. Is there a difference in the number of men who drink and drive compared to women? Why is there a difference?
12. At what point has someone had too much to drink that they should not be driving?
    a. What physical symptoms would someone have who has had too much to drink to be able to drive?
