# Supplementary material for: Sex differences in alcohol use patterns and related harms: A mixed-methods, cross-sectional study of men and women in northern Tanzania
Source: PLOS Glob Public Health. 2024 Nov 21;4(11):e0003942. doi: 10.1371/journal.pgph.0003942 (PMC11581317; doi:10.1371/journal.pgph.0003942)
Supplement: S2 Appendix — (PDF) [file pgph.0003942.s004.pdf]

S2 Appendix

COREQ (Consolidated criteria for Reporting Qualitative research) Checklist

A checklist of items that should be included in reports of qualitative research. You must report the page number in your manuscript where you consider each of the items listed in this checklist. If you have not included this information, either revise your manuscript accordingly before submitting or note N/A.

Topic	Item No.	Guide Questions/Description	Reported on Page No.
**Domain 1: Research team and reflexivity**			
Personal characteristics			
Interviewer/facilitator	1	Which author/s conducted the interview or focus group?	Included
Credentials	2	What were the researcher's credentials? E.g. PhD, MD	Included
Occupation	3	What was their occupation at the time of the study?	Included
Gender	4	Was the researcher male or female?	Included
Experience and training	5	What experience or training did the researcher have?	Included
Relationship with participants			
Relationship established	6	Was a relationship established prior to study commencement?	Included
Participant knowledge of the interviewer	7	What did the participants know about the researcher? e.g. personal goals, reasons for doing the research	Included
Interviewer characteristics	8	What characteristics were reported about the inter viewer/facilitator? e.g. Bias, assumptions, reasons and interests in the research topic	Included
**Domain 2: Study design**			
Theoretical framework			
Methodological orientation and Theory	9	What methodological orientation was stated to underpin the study? e.g. grounded theory, discourse analysis, ethnography, phenomenology, content analysis	Included
Participant selection			

Sampling	10	How were participants selected? e.g. purposive, convenience, consecutive, snowball	Included
Method of approach	11	How were participants approached? e.g. face-to-face, telephone, mail, email	Included
Sample size	12	How many participants were in the study?	Included
Non-participation	13	How many people refused to participate or dropped out? Reasons?	Included
Setting			
Setting of data collection	14	Where was the data collected? e.g. home, clinic, workplace	Included
Presence of non participants	15	Was anyone else present besides the participants and researchers?	Included
Description of sample	16	What are the important characteristics of the sample? e.g. demographic data, date	Included
Data collection			
Interview guide	17	Were questions, prompts, guides provided by the authors? Was it pilot tested?	Included
Repeat interviews	18	Were repeat inter views carried out? If yes, how many?	N/A
Audio/visual recording	19	Did the research use audio or visual recording to collect the data?	Included
Field notes	20	Were field notes made during and/or after the inter view or focus group?	Included
Duration	21	What was the duration of the inter views or focus group?	Included
Data saturation	22	Was data saturation discussed?	
Transcripts returned	23	Were transcripts returned to participants for comment and/or	Included

**Topic**	**Item No.**	**Guide Questions/Description**	**Reported on Page No.**
		correction?	

**Domain 3: analysis and findings**			
Data analysis			
Number of data coders	24	How many data coders coded the data?	Included
Description of the coding tree	25	Did authors provide a description of the coding tree?	Included
Derivation of themes	26	Were themes identified in advance or derived from the data?	Included
Software	27	What software, if applicable, was used to manage the data?	Included
Participant checking	28	Did participants provide feedback on the findings?	Included
Reporting			
Quotations presented	29	Were participant quotations presented to illustrate the themes/findings? Was each quotation identified? e.g. participant number	Included
Data and findings consistent	30	Was there consistency between the data presented and the findings?	Included
Clarity of major themes	31	Were major themes clearly presented in the findings?	Included
Clarity of minor themes	32	Is there a description of diverse cases or discussion of minor themes?	Included

Developed from: Tong A, Sainsbury P, Craig J. Consolidated criteria for reporting qualitative research (COREQ): a 32-item checklist for interviews and focus groups. *International Journal for Quality in Health Care*. 2007. Volume 19, Number 6: pp. 349 – 357
