# Supplementary material for: Sex differences in alcohol use patterns and related harms: A mixed-methods, cross-sectional study of men and women in northern Tanzania
Source: PLOS Glob Public Health. 2024 Nov 21;4(11):e0003942. doi: 10.1371/journal.pgph.0003942 (PMC11581317; doi:10.1371/journal.pgph.0003942)
Supplement: S4 Appendix — (PDF) [file pgph.0003942.s006.pdf]

S4 Appendix

Figure S1. Rationale for two clusters from k-means.
In order to find the optimal number of clusters for the k-means algorithm, we performed 29 methods and chose the number of clusters based on the agreement between them. These methods were the following: Elbow, Silhouette, KL, CH, DB, Duda, Pseudot2, Beale, Ratkowsky, PtBiserial, McClain, Dunn, SD index, Tracew, Friedman, Ball, Hartigan, Scott, Marriot, trcovw, Rubin, Ellipsoidal mixture model (equal volume and equal shape; EEV), Frey, Gap Maechler, CCC, Gap Dudoit, C index, sdbw, and Ellipsoidal mixture model (equal volume, shape, and orientation; EEE). All of these methods were implemented in the R package *NbClust* (44). The idea of a consensus-based decision was proposed by Lüdeck et al. (2020) (43) and was implemented in the R package *parameters*, precisely in the function *n_clusters*. A full description of each method can be found elsewhere (44).

For the community and patient sample, the 13 methods which agreed with each other in the decision of two clusters as the optimal number of clusters were the following: Elbow, Silhouette, KL, CH, DB, Duda, Pseudot2, Beale, Ratkowsky, PtBiserial, McClain, Dunn, and SD index.

Since two clusters were estimated for both samples, we can observe the distribution of observations across the clusters in Figure SX. To build this figure, Principal Component Analysis (PCA) were performed to create two main components based on the variables used to estimate the clusters (i.e., AUDIT and the dimensions of DrInC). In the community sample, people with lower scores in the Dimension 1 were grouped into the Cluster 2, which means that they showed similar values for alcohol-related variables (i.e., higher scores for AUDIT and the dimensions of DrInC). In the patient sample, higher scores for Dimension 1 represent those who scored higher for alcohol-related variables and were grouped into the Cluster 2 in the k-means analysis.
