# Supplementary material for: Sex differences in alcohol use patterns and related harms: A mixed-methods, cross-sectional study of men and women in northern Tanzania
Source: PLOS Glob Public Health. 2024 Nov 21;4(11):e0003942. doi: 10.1371/journal.pgph.0003942 (PMC11581317; doi:10.1371/journal.pgph.0003942)
Supplement: Attachment — Submitted filename: Response to email.pdf [file pgph.0003942.s007.pdf]

1. Before we proceed, we kindly ask that you address the discrepancy in the ethics documentation. The individual Mark Mvungi named as investigator on the ethics documents is not an author on the present submission. As we would generally expect the investigator named on the ethical approval to be an author on the submission, can you please provide an explanation for this situation?

Yes, thank you. Dr. Mark Mvungi was the original investigator on this study and he retired. He does not meet authorship criteria for this manuscript.

2. During our evaluation of the documents provided, we noted that your ethics approval letter did not cover the entire date range for participant recruitment. Before we can proceed further with the submission, please provide the original ethics document from Tanzania (of year 2015). If the document is in another language, please also provide an English translation. Please note that if this document is not included when your manuscript is resubmitted, it may be rejected.

We have uploaded ethics documents to cover the entire date range. Our apologies for this error.
