# Supplementary material for: Sex differences in alcohol use patterns and related harms: A mixed-methods, cross-sectional study of men and women in northern Tanzania
Source: PLOS Glob Public Health. 2024 Nov 21;4(11):e0003942. doi: 10.1371/journal.pgph.0003942 (PMC11581317; doi:10.1371/journal.pgph.0003942)
Supplement: Attachment — Submitted filename: 23April2024_Response to reviewers.pdf [file pgph.0003942.s008.pdf]

Dear reviewers,

Thank you kindly for taking the time to review and suggest improvements for our article. We have addressed all the comments raised in an effort to adequately revise this manuscript for publication at PLOS Global Public Health. This letter contains point-by-point responses to each comment.

Reviewer #1

This manuscript is very well written and highlights an important topic of alcohol use among women in Tanzania. The contribution is novel, and the combination of quantitative and qualitative analysis is unique. My only comment is that I think the discussion could benefit from a very brief expansion of the clinical implications (i.e., perhaps treatment implications, given that women are discouraged to seek care, and that women are often encouraged to use alcohol by men).

Thank you for this thoughtful comment. We have, as you suggested, expanded on the clinical implications of each component of our discussion to highlight these components.

Reviewer #2

The present manuscript uses mixed-methods to explore sex differences in alcohol-use and related harms within clinical and community settings in northern Tanzania. Strengths of this manuscript include the large sample, the clinical and community sample, and the mixed-methods approach. The paper is well-written and important for understanding patterns of alcohol use in this context. The findings of this study can be used to inform future alcohol interventions (and specifically, how to tailor such interventions to men versus to women), and also suggest what future research may be needed (i.e., to further explore if women are discouraged from seeking injury care due to alcohol use). While I believe that this manuscript should ultimately be published, I recommend that the authors address the following minor comments before it is accepted.

Thank you for your comment. We highly appreciate the time and effort spent reviewing our article, and we address each of your suggestions below:

After reading the full manuscript, it was unclear to me the rationale for including an injury patient's sample and a community sample. The author should elaborate on the specific rationale for these two samples (and why they were examined differently).

We are happy to clarify. Our previous studies of injury patients identified significant harmful alcohol use among men (doi: 10.1186/s12889-018-5144-z). In this study, the rationale for a community sample was to identify and describe populations with harmful alcohol use who are not presenting to the emergency department for care. We added this in Methods under the Study Design subsection. For example, in terms of sex, we sought to determine whether women with harmful alcohol use are not accessing the health system via the emergency department, or harmful alcohol use is simply not as common / not an identified need among women in the community, or another possibility for their lack of representation in injury patient samples.

Further, what was the rationale for including any injury patient versus patients who had injuries or other conditions likely related to alcohol? Was whether or not the patients' injury was related to alcohol recorded (and if so, how was this determined)?

This study was part of a larger award (see your question regarding this for more detail) to determine the proportion of injuries related to alcohol and the increased risk of injury due to alcohol among injury patients seeking care at the emergency department. Injury and alcohol were analyzed in a previous manuscript (doi: 10.1186/s12889-018-5144-z). To answer your question, though, an injury was determined to be related to alcohol if the patient had a blood alcohol content ≥.01% by breathalyzer and/or self-reported alcohol use during three specific time periods (within 6 h of injury, 24–30 h before injury, and 1 week before injury).

The authors only mention compensation in relation to the focus group discussions (line 186), and do not provide further information on this topic. The authors should clarify (a) in which parts of the study participants were compensated (e.g., just focus groups, surveys and focus groups, etc.,); (b) the amount participants were compensated; and (c) how this compensation amount was determined.
We have added the following information to the manuscript. Survey participants were given a small token of appreciation equalling about 2000 TSH or 1 USD. Focus group participants were provided with food and beverage for their participation. These compensation were determined by common practice at the research site and discussions with KCMC Ethics Committee and the Duke IRB.

The authors mention that the focus group discussions took about 45 – 60 minutes. However, they do not include how long the quantitative surveys took. The authors should include this information.
Thank you for pointing this out. The quantitative surveys took 30 to 40 minutes on average. We have added this information.

The authors mention that participants were screened for surveys/ focus groups and list information on eligibility criteria. However, they do not provide information on the number of people screened for each part of the study, how many screened were ineligible, and why they were ineligible. It would be helpful if the authors could include this information to help the reader better understand who was and was not included in this study.
Unfortunately, we do not have this information. For the community participants, the eligibility criteria was broad (age 18 or older and fluent in Swahili) and a convenience sample. Participants would only have been excluded if they declined to participate. For the injury patient sample, it would be helpful to know the number of patients indisposed due to severe injuries or illness, but we simply do not have this information and have added this to the Limitations section.

Regarding methods for the focus group interviews, it would be helpful if the authors could provide the following information:

A) What was the rationale for who was chosen to participate in focus group discussions? Further, what was the rationale for including more patients/patient relatives than community members? Were those who took part in the focus group discussions also participants in the quantitative surveys?
Thank you for asking for clarification. We have reworded / added information to make this clearer in the manuscript. After the quantitative surveys, a subset of participants were approached to participate in focus group discussions. The rationale for more patients and patient relatives than community members was to understand the direct lived experience with harmful alcohol use. The rationale for including two community member focus groups was to understand the broader social context regarding alcohol use from peoples who might or might not have lived experience.

B) What was the process for translating focus group discussions from Swahili to English? Was there an additional process for checking the translations?
Focus group discussions were translated by bilingual English/Swahili research assistants, and reviewed by a second bilingual research assistant.

C) Was NB the only person going through the full transcripts to code them (and presenting quotes to the full research team), or did a second person also go through them to ensure no quotes were missed? What was the rationale for coding the discussions in this way?
Thank you for raising this question. We have clarified the analysis approach as follows: "Full focus group transcripts were analyzed previously via an inductive thematic narrative approach (see El-Gabri et al. 2020 for primary analysis details) (45). As this manuscript concentrates on sex differences, we conducted a secondary analysis utilizing the previously developed codebook. NB deductively selected codes from this codebook that were responses to gender-specific questions in the FGDs, as well as quotes from the transcripts that spoke about sex-specific behaviors in response to more general questions.

The gender-specific codes were discussed and reviewed with the rest of the research team. Themes that were further analyzed for sex-specific differences included: alcohol initiation, consumption patterns, risk behavior, social stigma, and traditional beliefs. Alcohol initiation included information about circumstances and age at which men are first introduced to alcohol. Consumption patterns consider circumstances in which women consume alcohol, as perceived by the participants. Risk behavior was specific to drunk driving and willingness and circumstances to engage. Social stigma related to the specific beliefs related to men and/or women and traditional beliefs included information on how they influence alcohol initiation and consumption patterns.

The codes were extracted and categorized on Google Sheets. AT reviewed the coding and discussed the coding sheet and any questions about the coded data with NB. NB then created category summaries to discuss with the full research team."

The authors mention Table 1 once on line 242. However, I could not find this table in the manuscript. Further, it was unclear from the sentence in line 242 what this table is supposed to show. The authors should ensure that they include Table 1 in the text.
Our apologies. We have added Table 1.

I also recommend that the authors include a demographics table to help characterize the community sample, the clinical sample, and focus group participants. If this is not Table 1, the authors should consider adding an additional table.
We have added Table 1 to help characterize the community and patient samples. Unfortunately, we do not have demographics for the focus group participants. It was not standard practice in our group at the time to collect this information. We have since reworked and updated our protocols to ensure we do so.

It would be helpful to know if this study was done as part of a larger study, or if this was a stand-alone study. It would also be helpful to know if the findings presented are secondary or primary results from the study.
The study was completed as part of a larger K award (K01TW010000) to better describe alcohol use in northern Tanzania culture and create an intervention for injury patients who have alcohol use disorders. The analysis conducted in this manuscript is secondary results to help identify potential populations with harmful alcohol use who do not access the health system via the emergency department.

The authors should include why the data is not available in the manuscript.
Data cannot be shared publicly because of confidentiality and privacy concerns surrounding the human participant data. Data are only available upon request as data transfer requires a written agreement approved by the National Institute for Medical Research (Tanzania). Data inquiries can be sent to Gwamaka W. Nselela at moc.liamg@41mailliwakamawg.

When reviewing Table 2, I found myself scrolling back to the cut-offs for the AUDIT and looking for information to help contextualize the DrinC to better understand the scores. It would be helpful if this was included in Table 2 so the reader does not have to scroll back. If there are general cut-offs or guidelines to help contextualize the DrinC, it would be helpful to include these. If there are not cut-offs/ guidelines, what were typical scores in previous studies? Having more information about what scores mean will be helpful in interpreting the information.
Good point. We have added the cut-offs for the AUDIT to Table 2. We have also added information to contextualize the DrInC scores to the Methods section as well as to Table 2.

It would be helpful to know the gender of the participant providing quotes in the focus group discussions.

Unfortunately, we do not have this information (see response to your Table 1 question). We agree that it would be helpful to know the gender of the participant. We have added this to our Limitations section.

The acronym "KCMC" is first used in line 108, but is not defined until line 120. This should be swapped.
This has been corrected.

The acronym "ED" is first used in line 108, but is not defined until line 167. This should be swapped.
This has been corrected.

We are grateful for the valuable contributions made, and are available for any additional suggestions or questions.

Regards,

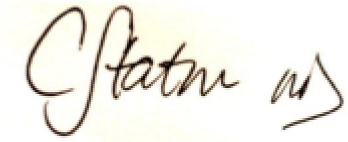

Catherine A. Staton, MD, MScGH
Corresponding Author (catherine.lynch@duke.edu)
Associate Professor of Emergency Medicine and Global Health
EM Director, Hubert Yeargan Global Health Residency and Fellowship Pathway
Director, Global Emergency Medicine Innovation and Implementation (GEMINI) Research Lab
Vice Chief of Faculty Development, Department of Emergency Medicine,
Duke University School of Medicine
Duke Global Health Institute
